# Bacterial risk factors for treatment failure and relapse among patients with isoniazid resistant tuberculosis

**DOI:** 10.1186/s12879-018-3033-9

**Published:** 2018-03-06

**Authors:** Phan Vuong Khac Thai, Dang Thi Minh Ha, Nguyen Thi Hanh, Jeremy Day, Sarah Dunstan, Nguyen Thi Quynh Nhu, Vo Sy Kiet, Nguyen Huu Lan, Nguyen Huy Dung, Nguyen Thi Ngoc Lan, Nguyen Thuong Thuong, Nguyen Ngoc Lan, Phạm Thị Thúy Liễu, Nguyễn Thị Hồng, Đào Công Điệp, Nguyễn Thị Kim Thanh, Nguyễn Văn Hội, Nguyễn Văn Nghĩa, Trương Ngọc Đại, Hoàng Quang Minh, Nguyễn Văn Thơm, Jeremy Farrar, Maxine Caws

**Affiliations:** 1 Pham Ngoc Thach Hospital, 120 Hung Vuong, Ho Chi Minh City, Vietnam; 2 Oxford University Clinical Research Unit, 763 Vo Van Kiet, Ho Chi Minh City, Vietnam; 3 University of Melbourne, Melbourne, Australia; 4 Liverpool School of Tropical Medicine, Pembroke Place L3 5QA Liverpool, UK; 5 Birat-Nepal Medical Trust, Kathmandu, Lazimpat Nepal

**Keywords:** Tuberculosis, Isoniazid, Resistance, Multidrug resistance, Treatment

## Abstract

**Background:**

Drug resistant tuberculosis (TB) is increasing in prevalence worldwide. Treatment failure and relapse is known to be high for patients with isoniazid resistant TB treated with standard first line regimens. However, risk factors for unfavourable outcomes and the optimal treatment regimen for isoniazid resistant TB are unknown. This cohort study was conducted when Vietnam used the eight month first line treatment regimen and examined risk factors for failure/relapse among patients with isoniazid resistant TB.

**Methods:**

Between December 2008 and June 2011 2090 consecutive HIV-negative adults (≥18 years of age) with new smear positive pulmonary TB presenting at participating district TB units in Ho Chi Minh City were recruited. Participants with isoniazid resistant TB identified by Microscopic Observation Drug Susceptibility (MODS) had extended follow-up for 2 years with mycobacterial culture to test for relapse. MGIT drug susceptibility testing confirmed 239 participants with isoniazid resistant, rifampicin susceptible TB. Bacterial and demographic factors were analysed for association with treatment failure and relapse.

**Results:**

Using only routine programmatic sputum smear microscopy for assessment, (months 2, 5 and 8) 30/239 (12.6%) had an unfavourable outcome by WHO criteria. Thirty-nine patients were additionally detected with unfavourable outcomes during 2 year follow up, giving a total of 69/239 (28.9%) of isoniazid (INH) resistant cases with unfavourable outcome by 2 years of follow-up. Beijing lineage was the only factor significantly associated with unfavourable outcome among INH-resistant TB cases during 2 years of follow-up. (adjusted OR = 3.16 [1.54–6.47], *P* = 0.002).

**Conclusion:**

One third of isoniazid resistant TB cases suffered failure/relapse within 2 years under the old eight month regimen. Over half of these cases were not identified by standard WHO recommended treatment monitoring. Intensified research on early identification and optimal regimens for isoniazid resistant TB is needed. Infection with Beijing genotype of TB is a significant risk factor for bacterial persistence on treatment resulting in failure/relapse within 2 years. The underlying mechanism of increased tolerance for standard drug regimens in Beijing genotype strains remains unknown.

## Background

Drug resistance in *Mycobacterium tuberculosis (M.tuberculosis*) is increasing worldwide and represents a major challenge to global TB control efforts. In 2015, there were 10.4 million new tuberculosis infections and 1.4 million deaths [1]. According to the World Health Organization (WHO), 17% of strains are now resistant to one or more of the major first line drugs [2]. Six percent of new TB cases and 20% of retreatment cases are multi-drug resistant (MDR TB) [2]. Multidrug resistant TB (MDR TB) is defined as resistance to at least isoniazid and rifampicin, the two most effective drugs in anti-tuberculous regimens. MDR TB must be treated with second-line drug regimens which are longer in duration, highly toxic, less potent and more expensive.

Rifampicin mono-resistance is rare and resistance to isoniazid is therefore the key precursor in the generation of MDR TB strains yet has received limited research attention. Ten percent of new TB cases and one third of retreatment cases globally are resistant to INH [2]. It is known from meta-analysis of trial data that treatment failure and relapse rates are higher among patients with undiagnosed INH resistance, yet the majority of these patients are still successfully treated using standard regimens [3–7]. The determinants of failure and relapse in those with INH resistance who fail despite good adherence to standard therapy are not well understood.

In Vietnam, as in most high burden settings, full drug resistance testing is only carried out for failure or retreatment cases and therefore isoniazid resistance is rarely detected in new TB cases. However, high treatment failure rates in isoniazid resistant TB cases may be fuelling the rise in MDR TB.

WHO has recommended that ethambutol (EMB) is added to the continuation phase for all new patients in countries with ‘high’ isoniazid resistance [8]. There is limited evidence for the efficacy of this regimen and the ocular toxicity of ethambutol will lead to cases of blindness in unnecessarily treated patients with drug-sensitive tuberculosis [9–13].

The study reported here aimed to determine [1] the proportion of failure/relapse in isoniazid resistant cases which is not captured by standard WHO monitoring [2] if bacterial factors can be used to predict those at highest risk of failure/relapse among those with isoniazid resistant tuberculosis and determine which patients would most benefit from modified regimens.

We assessed bacterial lineage, resistance mutation, isoniazid minimum inhibitory concentration (MIC), and additional drug resistance in 239 patients with INH resistant tuberculosis identified among a cohort of 2091 new smear positive pulmonary TB patients. INH resistant TB included all isolates resistant to INH but susceptible to rifampicin (ie. Not MDR TB). Patients with INH resistant TB were followed for 2 years to determine the rate of relapse/failure which is not detected by standard WHO criteria for TB treatment outcome classification.

## Methods

### Ethics

The study was approved by the Institutional Research Board of Pham Ngoc Thach Hospital as the supervisory institution of the district TB Units (DTUs) in southern Vietnam, Ho Chi Minh City Health Services and the Oxford University Tropical Research Ethics Committee (Oxtrec 030–07). Written informed consent was obtained from all patients prior to screening for INH susceptibility and, if resistant by MODS testing, a second written informed consent was obtained for enrolment into the INH study.

### Recruitment

**Fig. 1:**
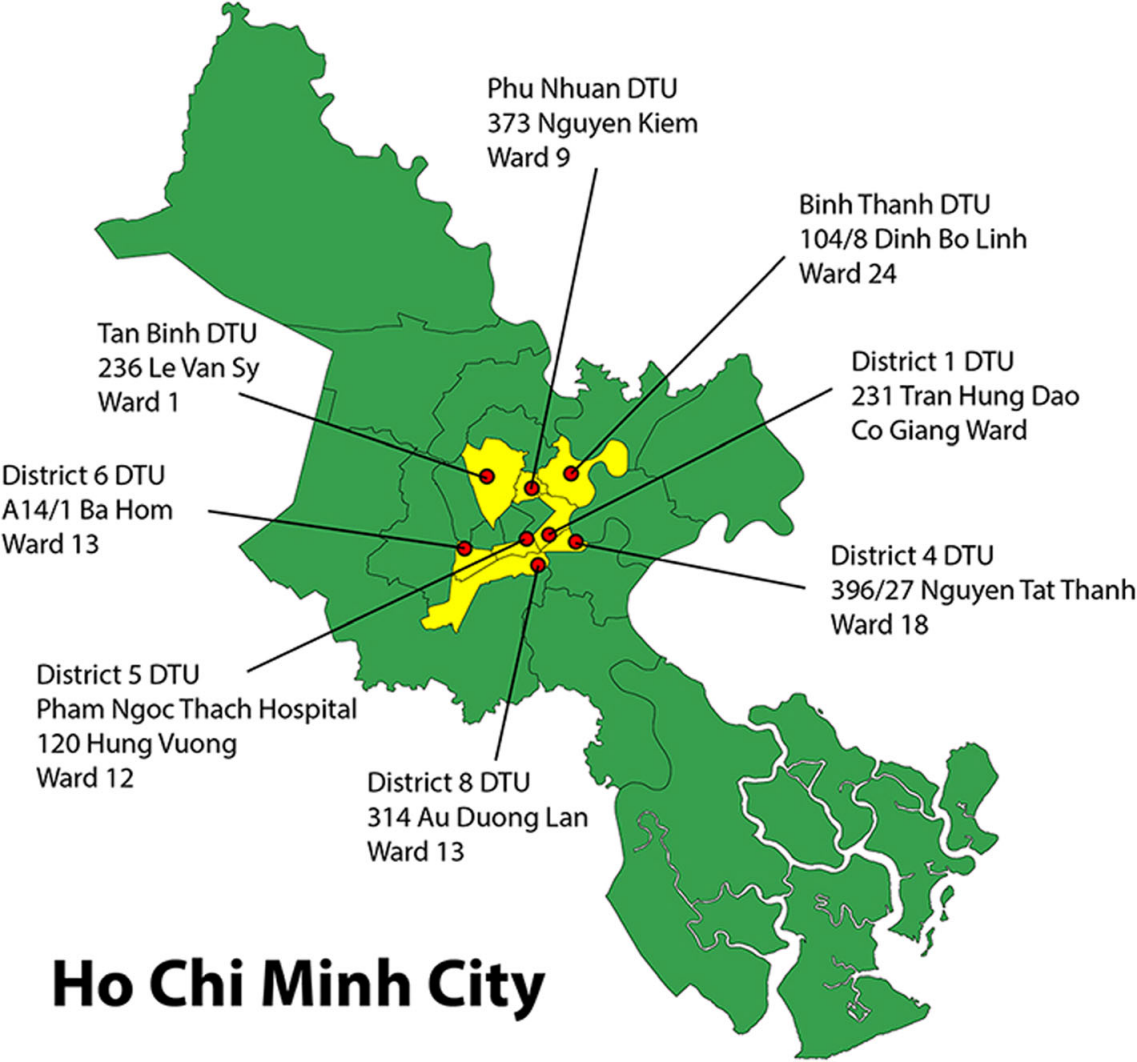
Map of geographical location of eight District TB Units in central Ho Chi Minh City participating in the study

HIV uninfected adult (≥18 years of age) patients with new smear positive pulmonary tuberculosis presenting at participating DTUs in Ho Chi Minh City were invited to participate. From December 2008 four DTUs (districts 4, 6, Binh Thanh and Phu Nhuan) and the out-patient department at Pham Ngoc Thach Hospital recruited patients. In October 2009, a further 4 DTUs joined the study (district 1, 3, 8 and Tan Binh). District 3 was withdrawn from the study due to low recruitment (9 patients) in early 2010. A map of participating DTU sites is shown in Fig. 1. The study completed recruitment at all sites in June 2011.

If written informed consent was given for INH susceptibility screening, a sputum sample was sent to the microbiology laboratory at Pham Ngoc Thach hospital for MODS testing. Patients with isoniazid susceptible tuberculosis by MODS were then followed up according to routine DTU practice using standard WHO outcomes (smear at 2, 5 and 8 months) and no further testing was conducted for the study. All patients with isoniazid resistant TB by MODS screening were invited to participate in the INH study, with more detailed follow-up and testing. A second written informed consent was obtained for recruitment to the INH study.

Inclusion criteria for screening were a negative HIV test, written informed consent, a positive sputum smear, no previous TB treatment, ≥18 years of age, not pregnant or planning to receive DOTS outside the participating study centres. Patients were eligible for inclusion in the extended INH study if they met the above criteria, gave written informed consent to the extended INH study and were infected by a strain resistant to INH on MODS screening.

### Treatment

Patients received treatment determined by the treating physician and usually in accordance with Vietnamese National TB Program (NTP) guidelines, which at the time of the study did not include any change in treatment for INH resistant TB unless the patient remained smear positive at 5 months. In accordance with NTP policy, patients smear positive at 5 months are classified as a treatment failure, tested for MDR TB and started on the retreatment regimen.

When the study commenced the standard regimen used in Vietnam was streptomycin (STR/S), rifampicin (RIF/R), INH (H) and pyrazinamide (PZA/Z) for the first two months (intensive phase) followed by 6 months with INH and EMB (continuation phase; 2SHRZ/6HE). In October 2009 the NTP replaced STR with EMB (2RHZE/6HE) as the first stage in a phased transition to the six month regimen (2RHZE/4HR) regimen. This enabled us to retrospectively compare failure and relapse rates between the two regimens (2SHRZ/6HE and 2RHZE/6HE) for patients with INH resistant TB but was not part of the original study design.

MODS was not accepted as a diagnostic assay for drug resistance within the NTP at the time of the study therefore patients with MDR TB diagnosed by MODS were referred to the NTP MDR treatment programme for confirmatory testing (using line-probe assay) and treatment according to standard NTP practice.

### Follow-up

All patients in the screening and INH study were followed up according to WHO guidelines with sputum smear evaluation at 2, 5 and 8 months (treatment completion). A positive sputum smear at month 5 or later was classified as a retreatment case and further managed according to standard NTP practice. In addition, patients in the extended INH study were further evaluated for symptoms and sputum culture at month 8, 12, 18 and 24. A positive smear or culture at month 5 or later was classified as a failure/relapse case. MDR patient outcomes were not followed-up.

### MODS testing

Drug susceptibility testing by direct MODS was performed according to the standard protocol for isoniazid and rifampicin [14]. Between December 2008 and 31 August 2010 the critical concentration for isoniazid was 0.4μg/ml. In 2010 the international recommended critical concentration changed to 0.1μg/ml following a meta-analysis by Minion et al. showing an increase in sensitivity compared to Lowenstein-Jensen (LJ) drug susceptibility testing (DST; 97.7% vs. 90.0%) [15] and we therefore adopted this change from 1st September 2010 until the end of the study (June 2011) [15].

### Bactec MGIT

Phenotypic drug susceptibility testing for STR, INH, RIF and EMB was performed for all isolates at completion of the study using Bactec MGIT SIRE at the OUCRU laboratory. MGIT DST results were used as the gold standard for confirmation of INH resistance in further analysis.

### Isoniazid MIC

INH antibiotic powder (Sigma, Germany) was used for determination of the INH MIC concentration at 0.2 μg/ml, 1 μg/ml, 2 μg/ml, 4 μg/ml, 8 μg/ml and 16 μg/ml using the 1% proportion method on LJ media.

### Genotyping

DNA extraction was performed using the CTAB (cetyl trimethylammonium bromide)/chloroform method [16]. Isolate lineage was determined by Large Sequence Polymorphism (LSP) typing for RD105, RD239 and pks 15/1, as previously described [17]. Isolates were categorised into one of 3 major lineages; Euro-American, East-Asian or Indo-Oceanic. Any isolates failing to generate PCR products for LSP typing were typed and classified by spoligotyping using the standard methods [18] and the international spoligotyping database [19].

### Statistical analysis

Treatment outcomes were categorized as favourable (cured and treatment completed) or unfavourable (death, treatment failed) according to WHO classifications. For INH resistant TB cases unfavourable outcomes included positive culture at month 5 or later.

INH-R TB was any *Mtb* resistant to isoniazid but susceptible to rifampicin (not MDR). Isolates in the INH-R cohort therefore included both INH monoresistant strains and strains resistant to INH and additionally resistant to streptomycin and/or ethambutol.

Multivariate logistic regression was used to identify variables associated with unfavourable outcome (death or treatment failure). A *P*-value of ≤0.05 was considered significant. All statistical analysis was done on Stata version 10 (Statacorp, USA).

## Results

### Treatment outcomes in screening patients

One patient Case Report Form (CRF) was irretrievable and therefore 2090 new pulmonary TB patients were evaluated by sputum smear microscopy after 5 and 8 months according to WHO guidelines followed by Vietnam NTP. The majority of patients (95.1%, *n* = 1987/2090) received the daily regimen according to Vietnamese NTP guidelines.

In total, 547 patients received 2SRHZ/6HE and 1440 patients received 2RHZE/6HE. The remaining 103 patients received alternative individualized regimens, such as 2SRHZ/RHZ/5HE, 2SRHZE/RHZE/5RHE, 3RHZE/5HE, 3RHZE/3RH, 2SRHZ/4RH and 2RHZE/4RH.

Patients treated at Pham Ngoc Thach hospital out-patient department were much more likely to receive individualized treatment regimens than those treated at the district TB units. Of those receiving individualized regimens, 95/103 (92.2%) were treated at Pham Ngoc Thach hospital out-patient department.

Overall, 1858 (88.9%) patients were cured and 6 (0.3%) patients were classified as ‘treatment completed’, therefore, 1864 patients (89.2%) were classified as having a favorable outcome. 16 patients died (0.8%), 121 (5.8%) failed treatment, 82 were lost to follow up (3.9%) and 7 (0.3%) were not evaluated. Therefore, a total of 226/2090 patients (10.8%) were classified as having an unfavourable outcome by WHO criteria.

### Comparison of treatment outcomes of two standardized treatment regimens (2SRHZ/6HE and 2RHZE/6HE)

The crude odds ratio (OR) for unfavourable outcome was 0.94 [95% CI 0.65–1.35, *P* = 0.723] for patients receiving 2RHZE/6HE compared to those receiving 2SRHZ/6HE. Adjusting for resistance to INH or MDR by MGIT DST did not substantially change the OR: adjusted OR = 1.09 [95% CI 0.71–1.66], *P* = 0.703.

#### MODS screening

MODS cultures were positive for mycobacteria growth in 1804 cases. Of these, 1407 cases (67.3%) were susceptible to both RIF and INH, 324 cases (15.5%) were resistant to INH and susceptible to RIF, 5 cases (0.2%) were susceptible to INH and resistant to RIF, 68 cases (3.3%) were resistant to both INH and RIF (MDR). There were 202 cases (9.7%) which had a negative MODS culture and 85 cases (4.1%) that were indeterminate. The indeterminate category included isolates for which there was a negative result in the MODS control well and/or contamination of the control or sample well.

The change of INH critical concentration, from 0.4 μg/ml to 0.1 μg/ml, increased the percentage of isolates determined as INH resistant by MODS from 17.4% to 21.2%.

The percentage of isolates resistant to INH by MODS which were confirmed by MGIT DST increased significantly following the concentration change; 88.3% (*n* = 197/223) for 0.4 μg/ml and 98.4% (*n* = 120/122) for 0.1 μg/ml (*P* = 0.0007).

Overall, there were 392 cases with INH resistance by MODS (324 cases which were rifampicin susceptible and 68 cases which were MDR). 50 (12.6%) cases declined to participate in the INH study. Therefore, 274 cases of INH resistant TB were eligible to enter the INH study.

### MGIT DST results

Further analysis of INH-resistant TB and MDR TB cases was based upon those with available MGIT DST as the gold standard (*n* = 1710, Table 1). Treatment outcome analysis included only those receiving standardised treatment regimens (*n* = 1623).

**Table 1:** Drug susceptibility results by MGIT testing of 1710 isolates

Resistance pattern	Number (%)
Fully susceptible	942
Streptomycin	285
Streptomycin, isoniazid	278
Izoniazid	94
Streptomycin, isoniazid, rifampicin, ethambutol	34
Streptomycin, isoniazid, rifampicin	33
rifampicin	7
ethambutol	2
Rifampicin, ethambutol	2
Isoniazid, ethambutol	4
Streptomycin, ethambutol	8
Streptomycin, rifampicin	6
Streptomycin, rifampicin, ethambutol	1
Streptomycin, isoniazid, ethambutol	12
Izoniazid, rifampicin	2
Total	1710

### Risk factors for INH resistant TB and MDR TB

Demographic risk factors for INH-resistant and MDR TB are shown in Table 2. Analysis of the association between bacterial lineage and INH resistant TB or MDR TB is shown in Table 3.

**Table 2:** Demographic risk factors for MDR and INH resistant TB

	MDR TB			INH Resistant TB		
	OR	95% CI	*P*-value	OR	95% CI	*P*-value
Male sex	1.00	0.54–1.85	0.993	1.05	0.80–1.38	0.710
Kinh ethnicity	1.10	0.34–3.59	0.870	1.35	0.78–2.35	0.282
Age category						
1: 18–25 years	Baseline					
2: 26–35 years	2.98	1.26–7.03	0.013	1.11	0.77–1.59	0.584
3: 36–45 years	1.38	0.54–3.55	0.499	1.07	0.75–1.53	0.715
4: 46–55 years-	1.26	0.47–3.33	0.648	0.79	0.54–1.16	0.229
5: ≥56 years	0.74	0.19–2.89	0.665	0.77	0.48–1.21	0.255
District						
Pham Ngoc Thach	Baseline					
Phu Nhuan	1.06	0.28–4.06	0.936	0.94	0.53–1.68	0.838
District 6	0.81	0.21–3.03	0.751	0.82	0.47–1.43	0.492
District 4	0.81	0.16–4.11	0.803	1.18	0.63–2.25	0.598
Binh Thanh	1.76	0.48–6.44	0.393	1.39	0.79–2.46	0.259
District 1	1.97	0.53–7.33	0.309	1.33	0.68–2.53	0.348
District 8	1.42	0.37–5.45	0.611	0.82	0.44–1.50	0.511
Tan Binh	0.60	0.12–3.02	0.535	0.76	0.40–1.45	0.405

**Table 3:** Association between drug resistance and bacterial lineage

Lineage^a^	Drug resistance pattern	Crude OR	95% CI	*P*-value	Adjusted OR^b^	95% CI	*P*-value
Beijing *n* = 1017							
INH resistant		1.45	1.15–1.81	< 0.001	1.44	1.15–1.81	**0.002**
MDR		2.59	1.44–4.63	0.001	2.54	1.42–4.57	**0.002**
Indo-Oceanic *n* = 406							
INH resistant		0.63	0.49–0.83	< 0.001	0.63	0.48–0.83	**0.001**
MDR		0.37	0.17–0.78	0.009	0.37	0.18–0.79	**0.010**
Euro-American *n* = 181							
INH resistant		0.88	0.62–1.25	0.477	0.89	0.62–1.26	0.504
MDR		0.36	0.11–1.17	0.091	0.37	0.11–1.19	0.094

^a^Comparison for each lineage against all other strains. Lineage was indeterminate (mixed lineage, failed PCR or DNA not available) for 106/1710 (6.2%) strains

^b^Adjusted for young age (< 35 years of age) and sex

Statistically significant findings are highlighted in bold

### Association between bacterial lineage and INH resistance mutations

Among 457 isolates resistant to INH by MGIT, Beijing lineage was significantly associated with *katG315* mutation among INH resistant isolates, (OR = 2.10 [95% CI 1.41–3.13], *P* = < 0.0001), while Indo-Oceanic strains were significantly less likely to have a *katG315* mutation (OR = 0.34 [95%CI 0.16–0.70], *P* = 0.003). No lineage showed an association with *inhA* − 15 promoter mutation. Indo -Oceanic strains were significantly more likely to be wild type, carrying neither a *katG 315* nor an inhA − 15 mutation.

### Treatment outcomes among INH resistant TB cases

239 patients in the extended INH study had confirmed INH resistant, RIF susceptible isolates by MGIT DST, received a standardised regimen and were therefore analysed for risk factors associated with unfavourable outcome. Using only routine programmatic sputum smear microscopy for assessment, 30/239 (12.6%) had an unfavourable outcome by WHO criteria. Thirty-nine patients were additionally detected with unfavourable outcomes during 2 year follow up, giving a total of 69/239 (28.9%) of INH resistant cases with unfavourable outcome by 2 years of follow-up.

#### Level of INH resistance (MIC)

LJ MIC results were available for 214/239 isolates. Among 239 isolates MIC for INH resistance were 0.2 μg/ml: 26, 1 μg/ml *n* = 81, 2 μg/ml *n* = 94, 4 μg/ml *n* = 8, 8 μg/ml *n* = 2 and 16 μg/ml *n* = 3.

MIC to INH was categorized into high (≥ 1 μg/ml) and low (< 1 μg/ml) for further analysis. Treatment outcomes (determined using smear and culture results) were evaluated by INH MIC level for 239 cases. There was no association between high MIC and unfavourable outcome (OR = 1.15 [95%CI 0.46–2.89], *P* = 0.764) or bacterial lineage (ANOVA, *P* = 0.289).

### Bacterial factors associated with unfavourable treatment outcome among INH resistant cases

Analysis of bacterial factors associated with treatment failure in isoniazid resistant TB cases is shown in Table 4. Beijing lineage was the only factor significantly associated with unfavourable outcome during 2 years of follow-up. (adjusted OR = 3.16 [1.54–6.47], *P* = 0.002.

**Table 4:** Analysis of bacterial risk factors associated with unfavourable outcome during 2-year follow-up in 239 INH-resistant TB cases

Risk factor	Crude OR	95%CI	*P*-value	Adjusted OR^a^	95% CI	*P*-value
Age < 35 years (*n* = 97)	0.77	0.43–1.38	0.383	0.71	0.39–1.30	0.267
Male sex (*n* = 177)	0.80	0.43–1.50	0.494	0.82	0.43–1.56	0.542
Ethambutol resistance (*n* = 7)	0.99	0.19–5.20	0.986	0.95	0.17–5.16	0.952
Streptomycin resistance (*n* = 185)	0.68	0.36–1.30	0.246	0.53	0.27–1.06	0.074
Treatment with streptomycin (*n* = 63)	1.21	0.65–2.25	0.558	1.28	0.67–2.45	0.449
INH MIC > 1 μg/ml (*n* = 107)	1.15	0.46–2.89	0.558	1.33	0.52–3.40	0.557
Resistance mutation						
KatG 315 (*n* = 173	0.90	0.47–1.71	0.738	0.91	0.46–1.79	0.775
InhA −15 (*n* = 18)	0.48	0.13–1.72	0.262	0.41	0.11–1.50	0.178
Wild-type (*n* = 49)	1.53	0.75–3.10	0.243	1.69	0.79–3.64	0.177
Lineage^b^						
Beijing (*n* = 163)	2.82	1.41–5.66	0.003	3.16	1.54–6.47	0.002
Euro-American (*n* = 31)	0.55	0.21–1.40	0.210	0.50	0.49–1.31	0.159
Indo-Oceanic (*n* = 30)	0.58	0.22–1.48	0.250	0.54	0.21–1.40	0.204

^a^adjusted for resistance to streptomycin and ethambutol

^b^6 isolates had an unclassified lineage

## Discussion

Approximately one third (28.9%) of INH-resistant TB cases which were susceptible to rifampicin had failed or relapsed treatment within two years of starting treatment. Importantly, only 12.6% (*n* = 30/239) were classified as unfavourable outcome using WHO criteria of sputum smear evaluation at 5 and 8 months and so the majority of these cases would have been classified as ‘cured’ and received no further evaluation under standard practice. Overall, the cohort of 2090 patients had treatment outcomes within the WHO targets for programmatic delivery which masked an unacceptably high failure/relapse rate among INH-resistant TB cases. It is possible that this undetected high relapse/failure is at least partly responsible for the relatively slow decline in TB incidence in Vietnam despite a comprehensive NTP delivering DOTS and exceeding WHO targets for over ten years. The addition of culture to evaluation detected 46 (19.2%) failures by 8 months, and a total of 69 (28.9%) cases by two years of follow-up. The regimens used by the Vietnamese NTP at the time of this study were inadequate for INH resistant TB and this is demonstrated by the high rate of unfavourable outcomes in this cohort. The regimen has now been changed in line with revised WHO recommendations in areas of high INH resistance to 2HRZE/4HRE [8]. However, there is no existing evaluation of this regimen and the study reported here raises the concern that current routine programmatic outcome surveillance may be inadequate to detect failure/relapse in patients with INH resistant TB.

The East Asian/Beijing lineage was confirmed to be associated with MDR TB and INH resistant TB in Ho Chi Minh City [17, 20–23]. This lineage was also shown to be associated with treatment failure in INH resistant cases. The reasons for this association with treatment failure are not clear and require further research of the pharmacodynamics associated with bacterial lineage to unravel the complexities of bacterial persistence during treatment. Understanding of persistor mechanisms in *M*.*tuberculosis* are thought to be the key to shortened treatment regimens after the failure of three novel short regimens containing fluoroquinolones for drug sensitive TB in 2014 (OFLOTUB (NCT00216385), REMOX TB (NCT00864383) and RIFAQUIN (ISRCTN44153044) studies) [24–26].

Importantly the MIC to INH was not a risk factor for treatment failure, suggesting the current critical concentration 0.2 μg/ml for resistance is appropriate to define clinical resistance with current INH dosages. Similarly, despite reported associations of katG315 mutation with higher MIC than inhA -15 [27–30], the mutation conferring resistance could not be used to determine those at high risk of treatment failure/relapse.

This study has several limitations in addition to the outdated regimen assessed. Due to financial constraints, there was no culture follow-up in the drug-susceptible arm and therefore we were unable to compare the rates of unfavourable outcome detected by enhanced evaluation in INH susceptible TB cases. The 30-month report of the landmark clinical trial ‘study A’ conducted by the International Union against tuberculosis and Lung diseases (IUATLD) showed an unfavourable outcome rate of 10% for fully susceptibe TB at 30 months using the 8-month regimen [31].

We did not determine if isolates from culture positive cases post-treatment were due to relapse or reinfection, beyond broad LSP typing, which is not sufficiently discriminatory to confirm relapse. However, the contribution of reinfection is likely to be small as HCMC is not an intensive transmission area and the annual infection risk is low. There is a possibility that cases were reinfected from an untreated original household source, but this would not in any case be detected by genotyping as the genotypes would be identical to the original infection event.

Changes in both the treatment regimen and MODS INH critical concentration were made during the study. Although outcome did not vary by treatment regimen, the change in MODS concentration resulted in an increased proportion of INH resistance detected in the second part of the study and therefore recruited into the intensive follow-up arm. We used MGIT DST to confirm susceptibility in analysed INH-resistant cases.

## Conclusions

Overall, these data confirm that the East Asian/Beijing lineage is associated with treatment failure in Vietnam. The data showing a third of those with INH resistant TB had unfavourable outcomes, the majority of which were not detected by standard WHO follow-up, suggest a rigorous evaluation of the new programmatic regimen (2HRZE/6HE) for areas with high INH-resistance is required. This should include symptom evaluation and mycobacterial culture where indicated up to 2 years to determine the true relapse and failure rate of the regimen in cases of INH resistance. Cost-effective solutions to early detection of INH resistant TB are a research priority, as are randomised controlled trials of alternative regimens for treatment of INH-resistant TB.

## Abbreviations

CRF: Case report form
CTAB: Cetyl trimethylammonium bromide
DTU: District TB Unit
EMB: Ethambutol
INH: Isoniazid
INH/H: Isoniazid
LJ: Lowenstein-Jensen media
LSP: Large sequence polymorphism
M. tuberculosis: Mycobacterium tuberculosis
MDR TB: multi-drug resistant TB
MIC: Minimum inhibitory concentration
NTP: National TB Programme
OR: Odds ratio
PZA: Pyrazinamide
RIF/R: Rifampicin
STR/S: Streptomycin
TB: Tuberculosis
WHO: World Health Organization
